# Targeting Groups Employed in Selective Dendrons and Dendrimers

**DOI:** 10.3390/pharmaceutics10040219

**Published:** 2018-11-08

**Authors:** Rodrigo Vieira Gonzaga, Soraya da Silva Santos, Joao Vitor da Silva, Diego Campos Prieto, Debora Feliciano Savino, Jeanine Giarolla, Elizabeth Igne Ferreira

**Affiliations:** Faculty of Pharmaceutical Sciences, University of Sao Paulo, Sao Paulo 05508-000, Brazil; gonzaga.rodrigo.v@gmail.com (R.V.G.); soraya.ssantos@yahoo.com.br (S.d.S.S.); vitorjvs@live.com (J.V.d.S.); diego.campos.prieto@gmail.com (D.C.P.); deborasavino@gmail.com (D.F.S.); jeanineg@usp.br (J.G.)

**Keywords:** dendrons, dendrimers, targeting groups, biomedical application

## Abstract

The design of compounds with directed action to a defined organ or tissue is a very promising approach, since it can decrease considerably the toxicity of the drug/bioactive compound. For this reason, this kind of strategy has been greatly important in the scientific community. Dendrimers, on the other hand, comprise extremely organized macromolecules with many peripheral functionalities, stepwise controlled synthesis, and defined size. These nanocomposites present several biological applications, demonstrating their efficiency to act in the pharmaceutical field. Considering that, the main purpose of this review was describing the potential of dendrons and dendrimers as drug targeting, applying different targeting groups. This application has been demonstrated through interesting examples from the literature considering the last ten years of publications.

## 1. Introduction

The drug targeting of cells, tissues or specific diseases is a powerful tool in the treatment of pathological disorders, since it may increase the chemotherapeutic effect and decrease the toxicity in normal tissues [1]. Dendrimers, on the other hand, have been extensively applied in this field, once drugs can be encapsulated inside them or conjugated in their surfaces through covalent bonds [1,2,3].

**Figure 1:**
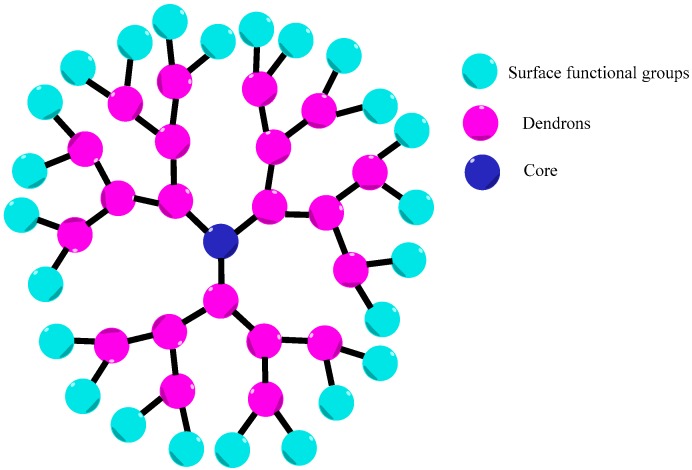
Dendrimer general structure.

Dendrimers represent an emerging class of low polydispersity hyperbranched macromolecules, which confer unique features such as: significant control over the molecular size, high branching density, nanoscale size and great surface functionality [4,5,6,7,8,9]. Those structures are composed of multifunctional core, which allows branches coupling, repeated branches layers from core named as dendrons and functional surface groups [4,10,11] (Figure 1). The first unity containing a core substituted with dendrons results in the first dendrimer generation. According to the increase of branches number in the dendrimer structure, higher dendrimer generations can be obtained. Therefore, the second layer of repeated units leads to the second dendrimer generation and thus subsequently [12]. Regarding to the dendrimer synthesis, these compounds can be, mainly, synthesized by convergent or divergent approaches [3].

Dendrimers present a great structural chemical diversity, as, for example: PAMAM (poly(amidoamine)), PPI (poly(propylenimine)), PLL (poly(lysine)), polyester, and PEHAM (poly(etherhydroxylamine)) dendrimers [7,12,13,14,15], among others.

Two features contribute to the dendrimer complexity, as generation number and surface terminal groups. In relation to generations, there are dendrimers from the first up to the tenth generation, although in the seventh-generation steric hindrance between the branches occurs, decreasing the synthetic yield of these compounds. Also, high generations may have influence in dendrimer toxicity [4,16,17]. In general, surface groups are anionic, cationic, or neutral and the toxicity studies have shown the cationic dendrimers as the most cytotoxic [14,18,19]. To overcome the cytotoxicity induced by dendrimers, diverse approaches have been developed based on suppression of the cationic surface through PEGylation, acetylation and chemical modification with anionic or neutral molecules [12].

Dendrimers and dendrons have several biomedical applications [18], especially as drug delivery systems [14,20,21,22,23,24,25,26,27,28,29,30,31,32,33]. Therefore, drug/bioactive agent may be either (1) loaded within the dendritic structure through electrostatic, hydrophobic, and hydrogen bonding interactions, or covalently conjugated to the dendrimer structure. Therefore, as drug/bioactive compound nanocarriers, dendrimers can provide controlled and/or targeted drug delivery either through encapsulating bioactive compounds [21,22] or (2) covalently conjugated to the dendrimer structure [34,35,36,37,38,39]. They present advantages as a well-defined chemical structure with low polydispersity and surface functional groups. In addition, dendrimers are stable compounds and their pharmacokinetics properties can be adjusted by controlling their size and shape [12].

It is also important to emphasize the multifunctional character of the dendrimers, which allow the linkage of different ligands of multiple receptors, achieving the selectivity and even synergistic effect [40].

Based on the foregoing interesting characteristics, this review aimed at describing the targeting groups employed in dendrimer and dendrons to obtain targeted drug delivery systems, which showed to be important in the field of pharmaceutical research. Table 1 reflects the targeting groups diversity related to the disease and the dendrimer architectures. 

### 1.1. Peptides as Targeting Groups

Some overexpressed enzymes in cells/tissues have been interesting by means of their specific peptides used as targeting groups to achieve selectivity. 

Cathepsin B, a lysosomal cysteine protease overexpressed in several cancer tissues, is known to degrade extracellular matrix during invasion and metastasis. Additionally, it is commonly observed as a prognostic factor in breast tumor. The tetrapeptide Gly Phe-Leu-Gly (GFLG) is a substrate of cathepsin B, which demonstrated good blood stability during delivery and allows intralysosomal drug release after endocytosis. PEGylated PAMAM dendrimers conjugated to DOX and GFLG spacer were described as an enzyme-responsive drug delivery system for breast tumor therapy. Peptide dendrimer–DOX compounds improved in vivo antitumor activity over commercial DOX formulation at the same dose. Additionally, the dendrimer provided lower toxicity as analyzed by acute changes in body weight, blood cell counts and histological analysis [81].

**Figure 2:**
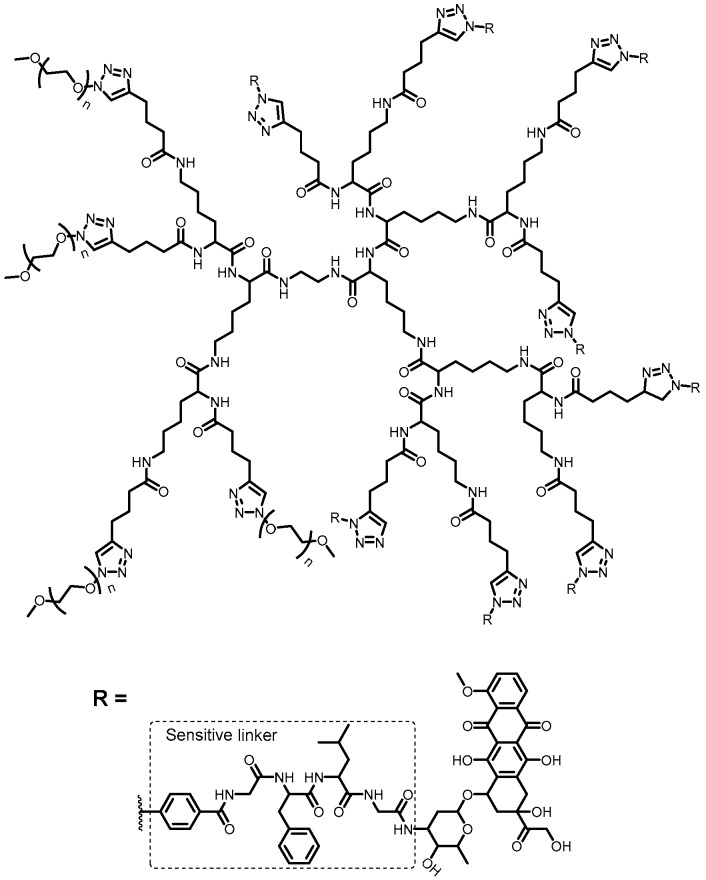
Structure of amphiphilic mPEGylated dendrimer-GLFG-DOX conjugate [50].

With the purpose of decreasing cytotoxicity and clearance, increasing selectivity and accumulating drug in breast tumor area, Li and coworkers [50] synthesized a dendrimer with two different dendrons (Figure 2). One of them, mPEGylated, was used to enhance molecular weight and size, decreasing renal filtration, thus accumulating it by enhanced permeability and retention (EPR) effect. Other PEGylated dendron, glycylphenylalanyl-leucylglycine conjugated with DOX (mPEGylated dendrimer-GLFG-DOX) is considered substrate for cathepsin. Through ex vivo studies, free DOX presented lower accumulation in tumor area as well as higher amount in other tissues such as in liver and kidneys. Meanwhile, the dendrimer was more accumulated in tumor area than in liver and other organs. In addition, they described this dendrimer as a possible control for tumor metastasis and in tumor growth inhibition, showing better effects than free DOX. The same group also used GFLG dendrimer, linking a targeting group conjugated to DOX (GFLG-DOX) on the surface. Applying the same strategy showed previously, the authors analyzed the dendrimers for ovarian tumor treatment, observing less activity than free DOX. Notwithstanding, a significant cytotoxicity against normal cell line was not observed at in vitro assays. On the contrary, at in vivo test, the dendrimer demonstrated higher anticancer efficacy than free DOX and could induce higher apoptosis levels. Histological analysis showed no toxicity and dendrimer presented more accumulation in tumor tissue [81].

GFLG dendrons conjugated to DOX were developed to self-assemble into nanoparticles. PEGylated dendron-GFLG-DOX demonstrated responsive-enzyme ability and, according to the in vitro assays, nanoparticles could kill breast cancer cells. Moreover, conjugated dendrimer was safer and showed higher antitumor activity than free DOX, due to enzyme-sensitive linker employed to compose PEGylated peptide dendron [50]. The same research group synthetized PEGylated PLL dendron, using Pro-Val-Gly-Leu-Ile-Gly (PVGLIG) peptide as spacer group, which is sensitive to matrix metalloprotease-2/metalloprotease-9, and was functionalized with DOX. This conjugated drug was evaluated for delivery in breast cancer cells through two mechanisms: increase of EPR factor due to PEG and via substrate enzyme-sensitive. The dendritic drug delivery system showed better biosafety, comparatively to free DOX, through in vivo assays. This compound exhibited higher in vitro cytotoxicity in breast tumor cells, in the presence of metalloprotease-2 enzyme. The association of peptide dendron PEGylation and enzyme-sensitive property revealed to be an efficient and safe possibility for drug delivery system. 

As reported in another work, a Janus peptide dendron-drug, composed by a sequence of PVGLIG peptide, sensitive to metalloproteases 2 and 9, and PEG, was synthesized [82]. Janus dendrimer, also called surface-block codendrimer, is a kind of compound containing a double-faced head with different properties [83]. DOX side effects with the dendrimer were lower comparatively to free drug administration. In addition, DOX activity was the same as the free drug after the complex administration 

Transferrin (Tf) is a targeting group for brain action, due to Tf receptor overexpression on the brain capillaries endothelial surfaces. Besides that, several cancer tissues overexpress Tf receptor on the surface of tumor cells, which provides iron to cells growth and participates in their survival [52,84]. HAIYPRH is a peptide with high affinity to Tf receptor and PEGylated PAMAM dendrimers were functionalized with it to deliver loaded DOX. The complex showed a high rate of internalization and selectivity, compared to free drug.

**Figure 3:**
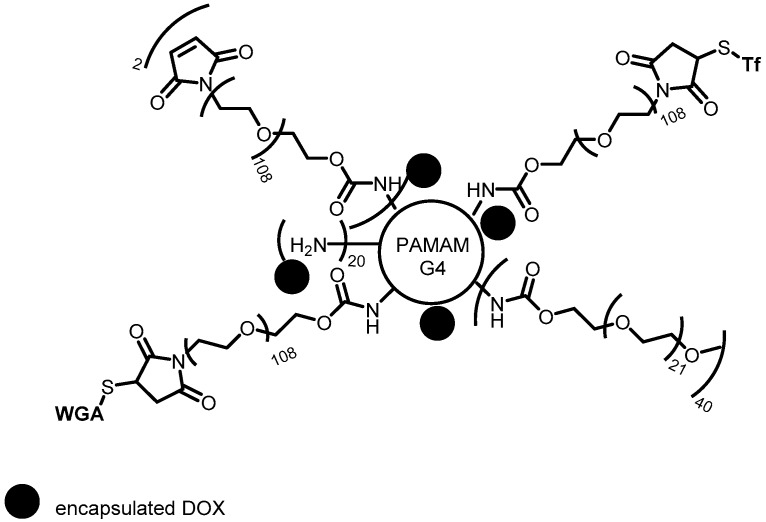
PEGylated dendrimers containing Tf and WGA (targeting groups) and encapsulated DOX for drug delivery to brain tumor [84].

Based on the same approach, wheat germ agglutinin (WGA) showed high affinity for the brain capillary endothelium, with high binding for tumor cells and reduced toxicity for normal cells. It was used so that PEGylated dendrimers containing both directing groups (Tf and WGA) and encapsulated DOX were designed to obtain targeting systems (Figure 3). Those dendrimers could cross blood-brain barrier and improve the cell uptake by brain tumor. The authors related the decrease of DOX cytotoxicity to the normal tissues, while it inhibited the growth of C6 glioma cells. Additionally, if there was DOX accumulation in tumor tissue, the inhibition rate to the C6 glioma cells increased, resulting in the blood-brain barrier transport improvement and synergistic effect of both endocytosis mechanisms (Tf and WGA) [84].

With the purpose of improving blood-brain barrier transport and drug accumulation in the glioma cells Li and colleagues [52] designed two dendrimer types based on G4 PAMAM dendrimer. One of them has DOX, PEG and Tf (G4-DOX-PEG-Tf) added on G4 PAMAM surface and the other has dual-targeting composed of DOX, PEG, Tf and tamoxifen added in G4 PAMAM surface (G4-DOX-PEG-Tf-tamoxifen). Dual-targeting dendrimer presented better transport ability in vitro blood-brain barrier trials. In addition, G4-DOX-PEG-Tf-tamoxifen had enhanced cytotoxicity against tumor cells and improved the drug delivery.

The association of chemotherapy and gene therapy is a promising strategy for treatment of cancer as, together, these techniques can provide synergic actions. Liu and colleagues [85] synthetized a copolymer with β-cyclodextrin (CD) core and poly(l-lysine) dendron (PLLD) to co-deliver docetaxel antitumor drug and MMP-9 siRNA plasmid for nasopharyngeal carcinoma therapy. MMP-9 siRNA was more effective for nasopharyngeal carcinoma therapy, when a folate modified (FA-CD-PLLD) was employed as targeting moiety. In addition, FA-CD-PLLD showed good blood compatibility and non-toxicity. According to the authors, this is a promising strategy for nasopharyngeal carcinoma therapy.

The use of Folic Acid (FA) as directing group is described in item 1.2, showing many interesting examples.

Lee and coworkers [3] also applied the tetrapeptide GFLG for anticancer drug targeting, aiming to evaluate if the tetrapeptide can deliver DOX to the site of action. The conjugate dendrimers presented superior cell uptake than free DOX in vitro trials, although the tumor growth inhibition was lower than the DOX alone. This might have happened due to the drug delayed release from the dendrimer. In vivo studies showed positive results such as high dendrimer concentration on the induced tumor, prolonged accumulation on the tumor site and low deposition in other organs. 

The protein αvβ3 integrin is overexpressed in melanomas, glioblastomas, and ovary cancer and, most importantly, it presents low expression in normal cells. The tripeptide arginine-glycine-aspartate (RGD) presents high interaction with cancer-related integrin and it is one of the most studied targeting group for cancer therapy. PAMAM (poly(amidoamine)) dendrimers coupled to the RGD peptide ensured the release of cytotoxic agents into the illness cells, showing no toxicity to normal ones [42]. The respective dendrimers were also covalently conjugated to RGD and they were applied as imaging agent for angiogenesis. 

Using the same approach, Ma and coworkers [45] conjugated PAMAM with RGD and encapsulated methotrexate (MTX) and observed this conjugate could reduce MTX toxicity mostly due to the slow drug release from the carrier. In vivo assays showed higher accumulation in tumor site, when compared to free MTX and non-functionalized dendrimer. Other examples comprehended Alexa Fluor 488, biotin or MTX connected to the branches. According to the authors, these findings could be a breakthrough development to delivery systems of multiple drugs and imaging agents.

Jiang and coworkers [43] developed a dual-targeting Janus dendrimer based on peptide dendrons for bone cancer. The branch was designed by peptide functionalized 5-fluorouracil (5-FU) and RGD (bone targeting group due to interaction with αvβ3 integrin receptor overexpressed in bone metastatic cells and osteoclasts). Four different dendrons that demonstrated binding ability to HAP (hydroxyapatite—inorganic component in hard tissues as bone and tooth) were synthesized. These target compounds could reduce toxicity in normal tissues and sustain the release. 

Other strategy to target bone tissue was to use poly aspartic acid (Asp_n_). In 2009, Ouyanga and coworkers [86] developed dendritic compounds with two or three fragments of Asp_(4–6)_ to increase the delivery of naproxen and improve its therapeutic index. All compounds presented good pharmacokinetic and pharmacodynamic properties, although the trimer had a slower binding rate than the dimer, due to steric hindrance. Other Janus dendrimers changed sequences of aspartic and glutamic acid aiming to deliver naproxen to the bone tissue with no significant differences [87].

**Figure 4:**
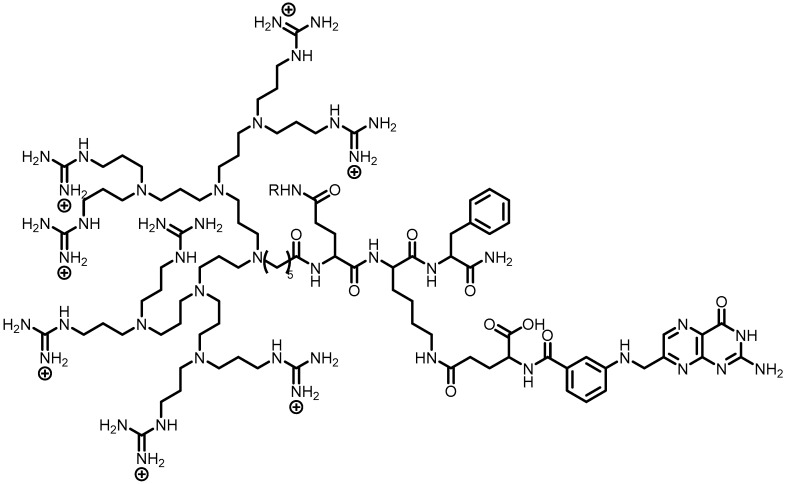
Chemical structure of G8-FKE-FA-DOX [44].

PPI (poly(propyleneimine)) dendrons were planned based on octa-guanidine residues as a molecular carrier. These compounds contained DOX, as well as lysosomal peptide to mimic cell-penetrating peptide features. The nanocarrier named as G8-PPI showed to be non-toxic and higher cellular uptake ability compared to arginine-octamer. It also exhibited excellent selectivity towards lysosomes in HeLa cells, being considered, therefore, an important candidate for targeting cancer therapy [46]. The same research group has developed dendron (G8-PPI) with FA, targeting group to folate receptor) and peptide FKE (Phe-Lys-Glu–substrate for cathepsin B overexpressed on neoplastic cells). G8-FKE-FA-DOX (Figure 4) demonstrated an excellent response to folate receptor-targeting, as well as increased cellular uptake and intracellular lysosome-mediated DOX delivery. In addition, G8-FKE-FA-DOX triggered the programmed cell death through extrinsic and intrinsic pathways, without affecting normal and folate receptor-negative cells [44].

Tat peptide, GRKKRRQRRRPQ, a transactivator of human immunodeficiency virus and a cell-penetrating peptide, can also be used to target cancer cells. G4 PAMAM dendrimer was conjugated to it to increase its internalization. Assays in heart, lung and spleen tissues showed that it presented low accumulation in healthy organs [88].

HER (Human Epidermal Growth Factor Receptor) is an important target for diverse types of cancer, being peptide H6 one of its ligands. In this context, PEGylated G4 PAMAM dendrimers were functionalized with peptide H6 to carry DOX for specific action in breast cancer cells. They did not show toxicity, while the DOX-encapsulated dendrimers presented high cytotoxicity [89].

Directed drug delivery system for pancreatic cancer was designed using tumor target peptide plectin-1, which is a biomarker for this kind of cancer, and siRNA. Nuclear receptor siRNA reduces the expression of antiapoptotic proteins, such as Bcl-2 and survivin. Consequently, tumor growth is not expected due to induction of apoptosis. The hydrophobic drug conjugated was paclitaxel and a synergistic effect was observed using siRNA. In vitro assays showed high dendrimers accumulation because of receptor-mediated endocytosis. Additionally, there was an increase in cell uptake and high transfection in Panc-1 cell lines [90].

The aptamer AS1411 is a selective oligonucleotide that binds to nucleolin, a nucleus membrane protein, which is overexpressed in some tumor cells, as gastric cancer. In general, aptamers are small single stranded RNA that can recognize and link, with high affinity, to other molecules by tridimensional folding [91]. Behrooz and colleagues [47] designed targeted polymers composed of PEGylated PAMAM dendrimers functionalized with aptamer AS1411 to deliver 5-FU and they succeeded. Based on the role of mucin on tumor growth and metastases [48] and in the importance of aptamers as targeting group, Masuda and coworkers [92] designed a sixth-generation glutamic acid modified with PLL dendrimer coupled to anti-MUC1 aptamer responsible for targeting several epithelial tumors. They observed that the dendrimer presented high cellular uptake and could be carried into the lysosomal and endosomal compartments. On the other hand, Taghdisi and coworkers [93] developed a polymer based on DNA dendrimer composed of MUC1 and AS1411 aptamers, employing the anticancer drug epirubicin, which shows cardiotoxicity and brown marrow suppression. Selectivity was achieved as the cellular viability assay demonstrated that normal cells were not affected, while tumor cells were destroyed after administration.

**Figure 5:**
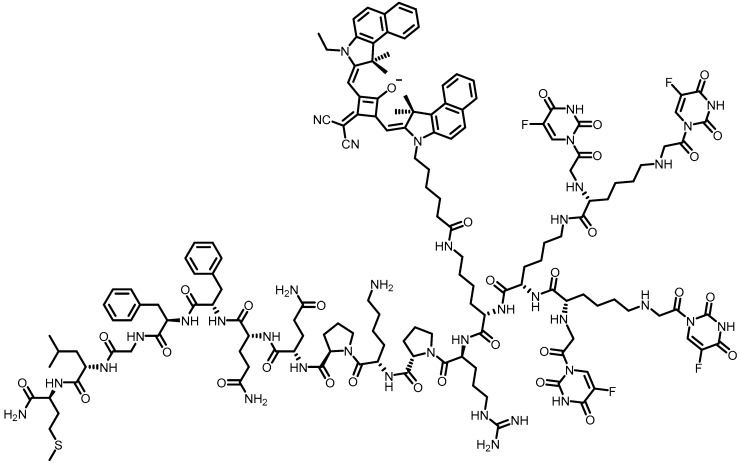
SP dendron containing branches conjugated to 5-FU for brain targeting [49].

Neurokinin-1 receptors, overexpressed in some cancer cells, are part of a family of undecapeptides of tachykinin neuropeptides. The substance P (SP) is rapidly internalized due to neurokinin-1 interaction. Therefore, Wu and coworkers designed a SP dendron with branches containing 5-FU and near-infrared labeled (Figure 5). SP dendron showed effectiveness in decreasing cell viability of tumor cells when compared to normal cells, which suggests an effective targeting feature [49].

The use of peptides as directing group for dendrimer nanocarriers for drugs has arouse increasing interest despite of their instability. The approaches herein discussed gave a panel of what can be done for achieving selectivity with these groups. 

### 1.2. FA as Targeting Group

**Figure 6:**
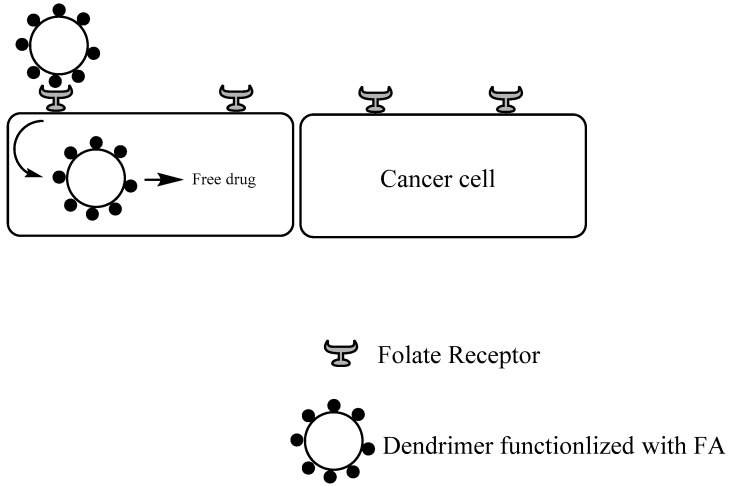
General targeting mechanism of FA-conjugated dendrimers.

FA is an important targeting group once folate receptor is overexpressed in diverse types of human carcinomas, such as ovary, colon, lung, and breast, being one of the most studied ligands. Folate receptor is a tumor marker that binds to folate-drug conjugates with high affinity, which can provide drug delivery by receptor-mediated endocytosis (Figure 6) [56,94,95].

FA-conjugated G5 PPI (poly(propylenimine) dendrimers were designed by loading DOX in the interior of dendrimer [53]. Other report showed DOX encapsulated in FA-G5 dendrimer and the DOX was released via photocleavable action [96]. Wang and colleagues [54], conversely, described G5 PAMAM dendrimers conjugated to FA, labeled with fluorescein isothiocyanate carrying 2-methoxyestradiol (2-ME), which led to cell death. Dendrimer release rate was more controlled than free drug in two pH conditions (7.4 and 5.0). No toxicity was observed in non-drug dendrimer and only 2-ME dendrimer was able to lower cell viability in KB cells. 

Experiments employing KB cells with overexpressed folate receptor, as well as KB cells containing normal FA receptor, demonstrated that 2-ME dendrimer was more recognized by cells with overexpressed FA receptor. This research group applied the same strategy for DOX, which showed sustained release without pH influence [97]. Similar approaches were used by Majoros and coworkers [55] and Shukla and coworkers [98]. Both studies used FA to deliver MTX to cancer tissues and the conclusions were the same: the conjugate was not toxic to healthy cells and caused cell growth inhibition. Other study performed by Singh and his group [99] aimed to synthesize PAMAM dendrimers with FA and PEG as targeting moieties. These dendrimers were further loaded with 5-FU to evaluate their capacity to specifically deliver this drug to cancer cells. PEG moiety increases the circulation time of the drug and the folate moiety delivers the 5-FU in a site-specific way in both receptor-mediated endocytosis and through EPR due to reduced lymphatic drainage. This effect occurs in most solid tumors, to increase the vascular permeability to provide nutrients and oxygen in tumor area for their growth [100,101].

**Figure 7:**
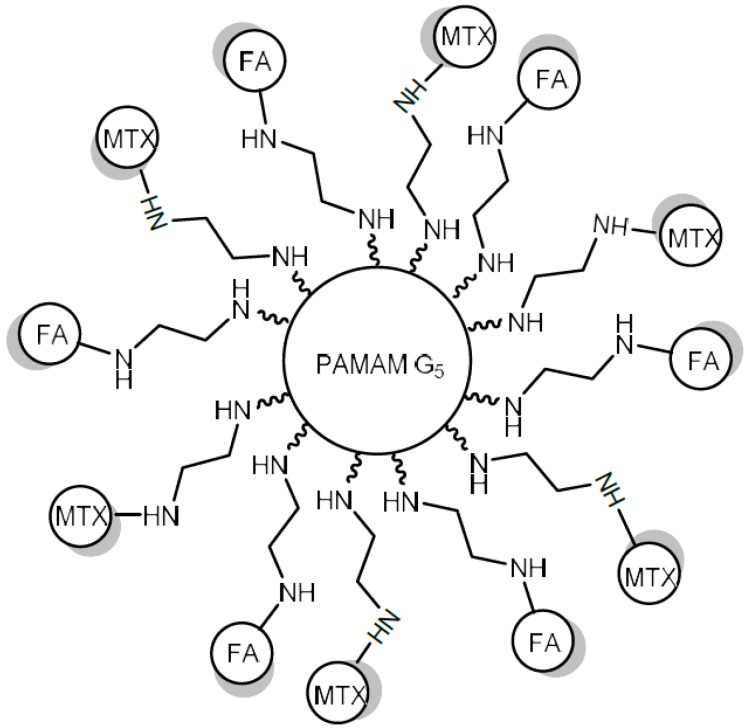
PAMAM G5 conjugated with FA and MTX [57].

Thomas and coworkers (64) studied FA as a directing group covalently coupled to G5 PAMAM dendrimers as a selective delivery system to the MTX. Another research synthesized G5 PAMAM conjugated to MTX (Figure 7) with the purpose of enhancing affinity by folate receptor [102]. MTX was employed for its dual activity, as a targeting and cytotoxic agent. Therefore, G5-MTX displayed better activity against tumor cells and promoted more effectively their death in contrast with free drug in in vitro tests [103]. Myc and colleagues [57] also designed G5 PAMAM coupled to MTX and FA to confirm their specificity and efficacy. In cytotoxicity assays, dendrimer was more efficient and presented higher action in cells with overexpressed FA receptor comparatively to normal cells, inhibiting the tumor growth.

It is important to notice that the conjugation of PAMAM with FA reduces the cationic toxicity of the dendrimer, as shown by Kersharwani and coworkers [58] in anticancer formulations in the drug targeting. 

PAMAM G3 and G5 containing FA and ursolic acid (UA-anticancer agent) were developed to overcome UA pharmacokinetic problems and provide the selectivity towards cancer cells. There was no difference regarding the release rate between G3 and G5 dendrimers. The findings suggested these compounds as good delivery systems [104].

**Figure 8:**
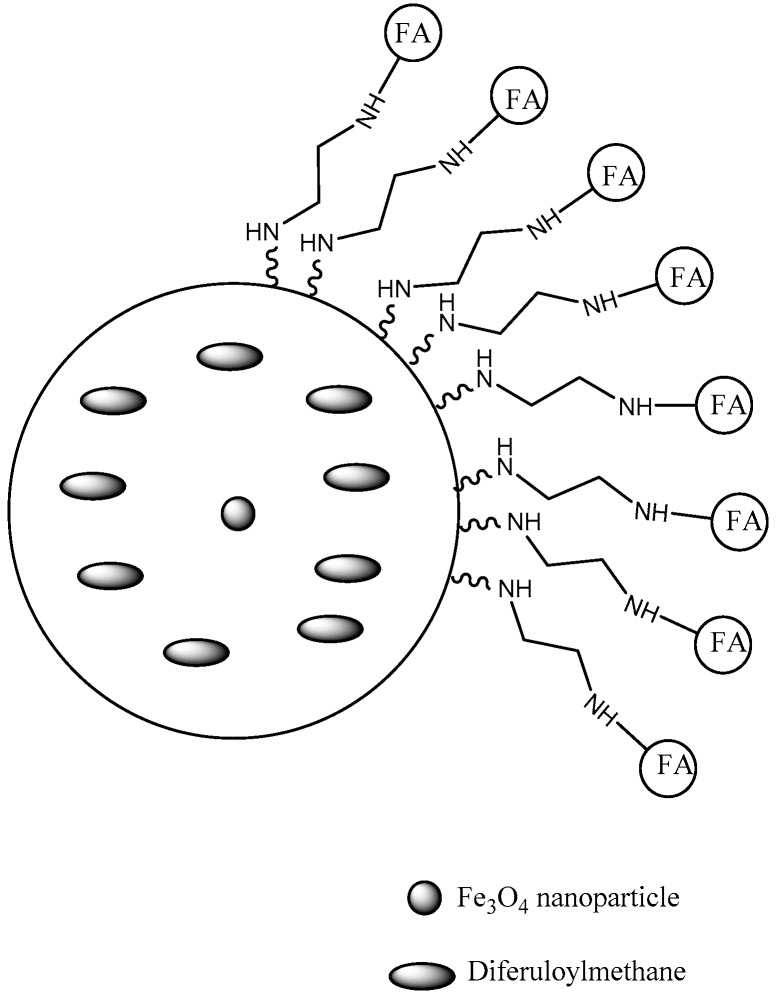
PAMAM dendrimer with SPION core encapsulated with diferuloylmethane and conjugated with FA [61].

FA was also conjugated to PAMAM dendrimers to load baicalin to improve water solubility and tumor selectivity. Even though this flavonoid presents anticancer effects, it displays low water solubility and bioavailability [105]. In another work, PAMAM dendrimers functionalized with FA were designed to deliver a highly hydrophobic flavonoid derivative, the 3,4-difluorobenzylidene diferuloylmethane. This study aimed to improve the water solubility and achieve the transport selectivity to overexpressed FA receptors in HeLa and ovarian cancer cells. Targeted dendrimers exhibited remarkable antitumor activity with greater accumulation in FA receptor-overexpressing cells, larger apoptosis rate, high expression of tumor suppressor phosphatase and tensin homolog, and inhibition of nuclear factor kappa B. All findings indicated the selective ability of this system [60]. The same research group developed PAMAM dendrimers composed of superparamagnetic iron oxide nanoparticle core (SPION), ornamented with FA on surface (FA-PAMAM) and containing 3,4-difluorobenzylidene diferuloylmethane via encapsulation to increase solubility and selectivity for ovarian and HeLa cancer cells (Figure 8). The compounds displayed a better anticancer action in targeted dendrimers than in non-targeted derivatives. Also, a larger population of cells suffering apoptosis due to upregulation of tumor suppressor phosphatase and tensin homolog, caspase 3, and inhibition of NF-κB were shown. In addition, these compounds have been studied as imaging agent in diagnostic, enhancing Magnetic Resonance contrast and fluorescence microscopy [61]. 

FA can also be used as directing group for inflammatory tissues. Indomethacin (anti-arthritis drug) was encapsulated in a G3.5 PAMAM dendrimer functionalized with PEG and FA. The study demonstrated the increase of plasma residence time of the complexes, as well as their higher concentration in inflamed tissue, reducing the stomach bleeding [62]. In other study four types of dendrimers were proposed contained different composition in terms of FA. The results suggested the folate amount provides an enhancement of the controlled delivery system. Indomethacin-FA-dendrimers increased plasma circulation time and reduced the cellular uptake by reticuloendothelial system [63].

PAMAM dendrimer and dendron have provided high ability for drug and gene delivery, exhibiting stability, and creating complexes with DNA. Dendron coated mesoporous particles have also been used for intracellular plasmid-DNA delivery. Mesoporous silica nanoparticles have attracted interest due to their multifunctional properties and have been studied as a template for drug delivery. Weiss and colleagues [64] investigated the application of mesoporous silica nanoparticles coated with PAMAM dendrons and FA for drug targeting to cancer cells. These dendrimers showed high loading capacity, low cytotoxicity, and redox-driven cleavage through disulfide bridges. Their targeting potential were able to enhance cellular uptake.

Magnetic resonance imaging agents were designed using G5 PAMAM dendrimer conjugated to FA to obtain targeted magnetic resonance imaging contrasts. These compounds were coupled to DOTA(1,4,7,10-tetraazacyclododecane-1,4,7,10-tetraacetic acid) chelator, forming stable complexes with gadolinium (Gd III). 3D Imaging assays in model murine of human cancer revealed the signal increase in tumors with targeted GdIII-DOTA-G5-FA, comparatively to the non-targeted GdIII-DOTA-G5 contrasts [106].

Another application of imaging agent using G3 PAMAM dendrimers was saccharide-terminated (d-glucohepton-α-1,4-lactone) functionalized to MTX, antifolate agent. Surface Plasmon Resonance studies indicated a three time increase recognizing G3-MTX by FA receptor in comparison to free FA [107].

Dendron micelles were developed for a drug delivery platform based on nanoparticles able to carry the drug into polyethylene glycol corona. The compounds were developed using various PEGs molecular weight to build the dendrons. Moreover, the conjugated constituents were varied to dendrons-FA and incorporated into dendron micelles, obtaining self-assembled nanostructure based on copolymers, containing an amphiphilic triblock. According to the authors, these compounds may be employed further to design efficient targeted nanocarriers for the treatment of several diseases [108].

Considering the number of examples of FA-conjugate dendrimers, briefly presented herein, it is clear the importance of using this directing group either in therapeutic agents or as imaging agents.

### 1.3. Carbohydrates as Targeting Groups

The use of carbohydrates is largely widespread in the research of drug targeting, considering the variety of receptors that can recognize them. As the kind of receptors changes from tissue to tissue, the targeting dendrimer containing carbohydrate may be more efficient [109]. The interaction with the carbohydrates in the membrane leads to selective internalization providing the carbohydrate receptor is specifically identified.

With this purpose, the asialoglycoprotein receptors (ASGPR) are highly employed as target. ASGPR are present on the surface of hepatic tumor cells, which allows the use of glycosylated nanocarriers for development of targeted drug delivery systems. *N*-acetylgalactosamine (NAcGal) is a selective sugar, substrate for ASGPR. Considering that, NacGal has been coupled to the G5 PAMAM dendrimers through peptide and thiourea bonds to act on ASGPR, being responsible for receptor-mediated endocytosis. These dendrimers functionalized with NAcGal were planned for drug targeting in hepatic cancer, aiming to compare the cell uptake with functionalized or non-functionalized dendrimer. According to the authors, NAcGal application is a promising strategy in drug targeting [65].

**Figure 9:**
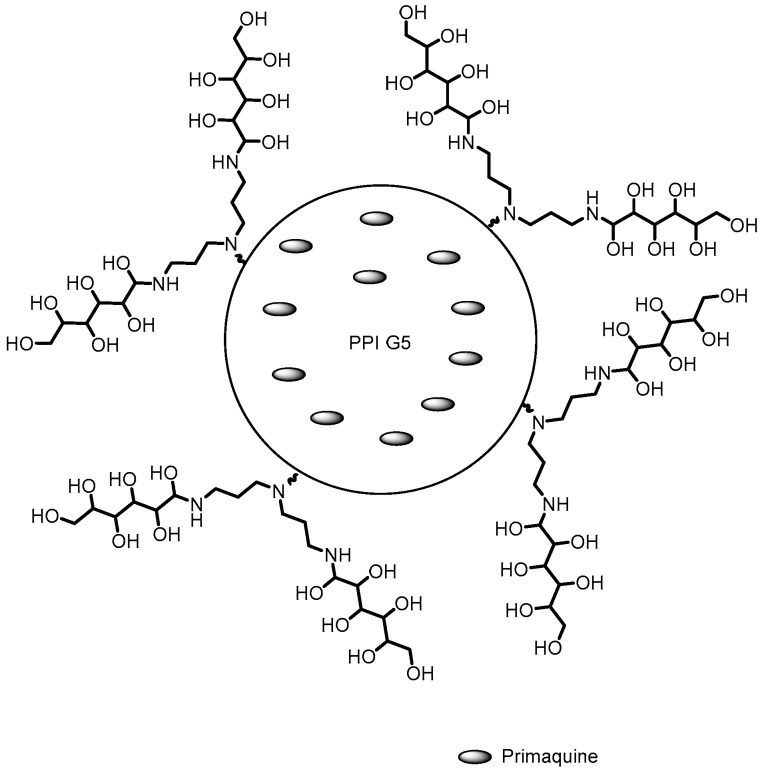
PPI G5 dendrimer conjugated with galactose and encapsulated with primaquine [66].

Another report showed the conjugation of galactose and DOX in PAMAM dendrimers to obtain a targeted drug for hepatoma cells [1]. Bhadra and coworkers [66] used galactose and primaquine conjugated with PPI dendrimer for malaria (Figure 9). The galactose conjugated dendrimer was able to decrease the hemolytic property of the primaquine and target the erythrocytes better than other evaluated nanoparticles.

Dutta and coworkers [67] designed dendrimers composed of mannosylated-PPI (MPPI) containing efavirenz and PPI-efavirenz to reach macrophages. Once HIV virus is inside these immune system cells, it is expected that the dendrimer cited above can be more efficient to combat it. Both dendrimers were able to decrease the drug toxicity. However, the mannose derivative presented 12-times-higher cellular uptake when compared with that of free drug and the dendrimer conjugate without the target carbohydrate. In another study, applying the same antivirus agent, MPPI and T-Boc-glycine-PPI (TPPI) dendrimers were described to decrease serum concentrations and drug side effects. Both dendrimers showed good results in cell uptake assays, since mannose interacted with lectin receptor and TPPI was absorbed via phagocytosis. The same group designed other two types of G5 PPI dendrimers as carriers with and without mannose (MPPI and PPI, respectively). MPPI showed more prolonged release ratio than PPI and, in in vitro cellular uptake assays, MPPI was more effective than free lamivudine and free dendrimer. Furthermore, significant improvement of anti-HIV activity was observed by MPPI when compared to the free drug, which could be related to the cellular uptake enhancement [109].

In other study, mannosylated PEGtide dendrons, G1 to G5, were synthesized containing amino acids, PEG and functionalized with mannose to reach macrophage. These compounds demonstrated good water solubility and potential biocompatibility due to high PEG in dendritic structure. Mannosylated dendrons presented higher uptake than non-mannose derivatives in murine models. Therefore, dendrons were mannose-dependent receptor for cell uptake. PEGtide dendrons could be an efficient platform to drug delivery and imaging applications [59].

**Figure 10:**
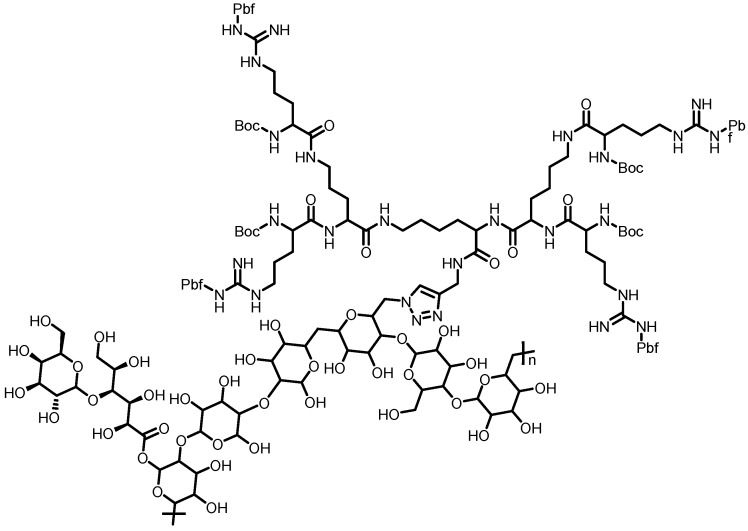
Chemical structure of LP-g-G3P an amphiphilic dendrimer [68].

Potential targeted drug delivery system was developed with arginine G3 dendron covalently attached to a hydrophilic polysaccharide (pullulan), which is a neutral linear compound. The LP-g-G3P is composed of lactosylated pullulan-graft-G3arginine dendrons, which showed self-assemble ability, as well as small size particles, low polydispertion and higher affinity to lectin receptor (Figure 10). DOX was encapsulated in LP-g-G3P through multiple interactions, was internalized into the hepatoma carcinoma cells, inhibiting cell proliferation. This type of targeted dendrimer showed to be a promising directed drug delivery system [68].

Blood-brain barrier membrane can overexpress several types of receptors and proteins responsible for transportation to the brain, as for example sialic acid receptors and glucose transporters. Patel and coworkers compared the efficiency in drug targeting of PPI dendrimers functionalized with sialic acid (SPPI), glucosamine (GPPI) and concanavalin A (CPPI). Paclitaxel was entrapped in dendrimer cavities. All derivatives exhibited lower hemolytic property than free drug. Moreover, the dendrimers presented a better accumulation in the brain than in other organs, such as liver and kidney when compared to free paclitaxel and PPI. For targeting potential, SPPI demonstrated the best results, implying the sialic acid receptor as a good strategy for drug delivery in central nervous system [69].

### 1.4. Monoclonal Antibodies as Targeting Group

Monoclonal antibodies developed against specific antigens may aid to target drug delivery to the site of action [70]. However, few examples have been found, considering the profile of these compounds, which could lead to many unwanted reactions in the body.

Prostate specific membrane antigen J591 antibody was conjugated to the G5 PAMAM dendrimer, being capable of selectively bind to the prostate specific membrane antigen receptor [70].

Interleukins have been employed in dendrimers functionalization for drug delivery of some diseases such as cancer, which can overexpress receptors for these molecules. Interleukin-6 (IL-6) is a crucial cytokine, which acts in angiogenesis, owing to fast tumor neovascularization. IL-6 was coupled to PAMAM dendrimer and the internalization and competitive assays indicated its fast and efficient cellular uptake. This molecule presented high affinity for HER, which resulted in significant internalization of IL-6-G5 PAMAM dendrimers into HeLa cells via receptor-mediated endocytosis. The same research group compared IL-6 and RGD functionalized PAMAM dendrimer to target HeLa cells. DOX was encapsulated inside the dendrimer and, then, its cellular uptake and in vitro toxicity were compared to free drug. Both functionalized dendrimers were less toxic than free DOX due to slow release of the drug from dendrimer, demonstrating better cellular uptake when compared to free drug [1].

### 1.5. Other Targeting Groups

Besides those groups described before, whose action has been evidenced by several studies, many different types of targeting group were found as potentially interesting with the purpose of selectively directing the drug action to specific cells/tissues.

Biotin is a micronutrient, which participates in fatty acid biosynthesis, gluconeogenesis, cell growth and catabolism. In addition, biotin level is rapidly increased in tumor cells, proving to be an interesting approach [1,71]. PAMAM dendrimers were functionalized with RGD peptide and then, biotinylated [110]. In another study, biotinylated G4 and G5 PAMAM were planned to overcome the blood-brain barrier. The uptake and selectivity in HeLa cells were more appropriate for biotinylated dendrimers and more selective for cancer cells, without toxicity [111]. Sodium-dependent multivitamin transporter has been indicated as responsible for biotin uptake. All findings imply that this is an interesting approach to improve therapeutic efficacy and decrease side effects of anticancer agents [1,110].

Other potential targeting group is the follicle stimulating hormone receptor, which is overexpressed by ovarian cancer cells. Taking this into account, Modi and colleagues [72] designed G5 PAMAM labeled with fluorescein and follicle stimulating hormone 33, since it presents high affinity to follicle stimulating hormone receptor. The dendrimer showed better cellular uptake profile than labeled dendrimer, mainly by respective receptors. 

Wen and colleagues [112] designed a nanoparticle conjugated with dendrons to deliver a photosensitizer. Natural nanoparticle *Cowpea mosaic virus* (CPMV) was used due to its target property. Additionally, CPMV has been shown to be selective for subpopulation of macrophages in cancer cells. The photosensitizer can react under light, resulting in reactive oxygen species, killing cells. Porphyrin is widely employed as photosensitizer, because it leads to electrostatic interaction with CPMV-dendron surface. The study succeeded in deliver the photosensitizer in the proper site.

It is important to consider the tendency of designing theranostic agents, that aggregates drugs and photosensitizers [113].

**Figure 11:**
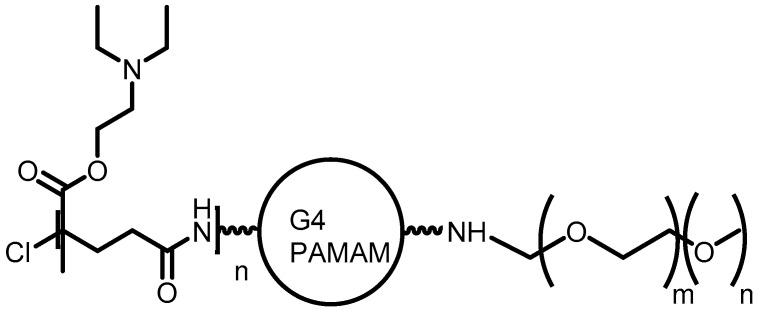
PAMAM dendrimer derivative, poly(2-(*N*,*N*-diethylamino)ethyl methacrylate) with methoxy-poly(ethylene glycol)-poly(amido amine) for 5-FU encapsulation [73].

Jin and colleagues [73] synthesized a PAMAM dendrimer derivative, poly(2-(*N*,*N*-diethylamino)ethyl methacrylate), with methoxy-poly(ethylene glycol)-poly(amido amine) loaded with 5-FU (Figure 11). The poly(2-(*N*,*N*-diethylamino)ethyl methacrylate derivative is a nanostructure sensitive to pH, from which 5-FU release is favored in the tumor acidic medium. This does not happen in the blood, due to the neutral/basic environmental characteristics. According to the authors, this system is a promising nanocarrier because it provides great drug encapsulation, high targeting, and fast drug release in tumor.

Polyethylene glycol (PEG) has been widely used in dendrimers with many purposes, as, for example, to confer biocompatibility through cytotoxicity and hemolytic toxicity reduction, improvement in water solubility, decreased particle aggregation and opsonization by the reticuloendothelial system and tumor accumulation increase by EPR as well [41]. The examples that follow present some of those applications.

Acid-sensitive bindings between drugs and PEGylated PAMAM dendrimers allowed drug release from polymer-drugs into the acidic cellular environment after tumor cell internalization, preserving the stable compounds in the bloodstream [114]. The first acid-sensitive bond polymer proposal was the *cis*-aconityl linkage in G4 PEGylated dendrimers, developed to obtain a selective drug delivery system for tumor action containing DOX. Therefore, the *cis*-aconityl acid-sensitive binding was introduced between DOX and the polymer carrier, resulting in PPCD (PEG-PAMAM-*cis*-aconityl-DOX conjugates). In addition, the researchers synthesized the acid-insensitive derivative composed by succinic bond, producing PPSD (PEG-PAMAM-succinic-DOX conjugates) for comparison. PPCD increased cytotoxicity in murine model of B16 melanoma cells, due to drug release in lysosomes after cellular uptake. PPSD derivatives released DOX in any pH condition showing low cytotoxicity in tumor cells. This evidenced the importance of acid-sensitive bindings as a targeted group. 

Super stealth liposomes with PEG-dendron-phospholipid using a β-glutamic acid dendron as an anchor to PEG attachment and several distearoyl phosphoethanolamine lipids were synthesized [74]. The liposomal composition demonstrated higher stability, lower toxicity, greater intracellular uptake, prolonged half-life time, improved biodistribution profile and enhanced DOX anticancer potency.

In the same way, a micellar drug delivery system was designed, containing dendrons conjugated to a hydrophilic PEG linear polymer of well-defined structure. Their biodegradable polyester dendrons were coupled to an antiangiogenic drug, combretastatin-A4, aiming to obtain proper sized flower-like hydrosoluble micelles for passive tumor targeting, enhancing the permeability and retention. The drug release from this conjugate occurred in acidic conditions, which is an interesting profile. In assays to evaluate the antiangiogenic efficacy, this dendrimer reduced the cell viability and uptake, showing efficient inhibition [75].

**Figure 12:**
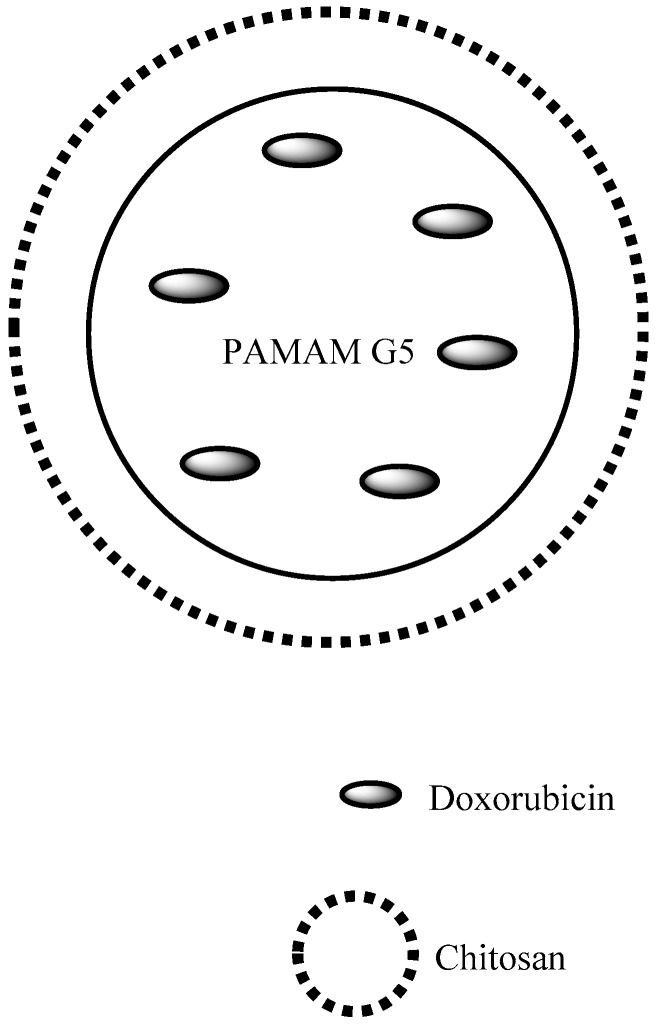
Nanocapsule of chitosan containing PAMAM G5 encapsulated DOX [76].

Another approach was based on the difference between physiological and tumor pH to lead a smart drug delivery system. In this context, Qi and coworkers [76] designed a dendrimer with carboxymethyl chitosan (CMCS) as shell and PAMAM as core, responsible for interacting via electrostatic adsorption. There was high drug release due to the positive charge in PAMAM surface masked by negative CMCS charge, decreasing dendrimer clearance and toxicity. Moreover, when dendrimer reached tumor area, CMCS became positively charged, leaving PAMAM surface, owing to pH difference. Through this approach, DOX was encapsulated in PAMAM (PAMAM-DOX-CMCS) and its rate release was correlated with the conjugate (Figure 12) increase, when pH dropped from 7.4 to 6.5 in 48 h, while free DOX was insensitive to pH. PAMAM-DOX-CMCS exhibited greater uptake than free DOX, indicating that CMCS was releasing from PAMAM surface at pH 6.5 and afterwards, through positive surface charge.

In previous studies, Li and coworkers [51] showed that supramolecular hybrid dendrimers exhibit 50,000 times more gene transfection efficiency to tumor cells than single dendrons. Taking this into account, they synthesized a supramolecular dendritic system composed of PLL and poly(l-leucine), which interacts through non-covalent bonds, leading to an amphiphilic self-assembly structure, mimicking a virus capsid. DOX was encapsulated in hydrophobic supramolecular dendritic pocket (D-CLNs) and at in vitro test, this molecular architecture disassembled, and drug was released.

Another way to target tumor tissue is through pH-sensitive compounds, using a group that links to the dendrimer via pH-sensitive bond, as, for instance, the hydrazone bond [115] or conjugating the respective drug in a G5 PAMAM with succinimydilpropylamine on the dendrimer surface [116]. They showed an interesting profile of selectively deliver DOX. In both cases, DOX release was dependent of pH, as proposed.

Also using a pH-sensitive hydrazine bond, a novel amphiphilic fluorinated peptide dendron functionalized with dextran was successfully synthetized. This conjugate has demonstrated self-assembly ability in carrying hydrophobic drugs. In in vitro assays, this dendron showed an excellent biocompatibility for normal and tumor cells, exhibiting a stimulus-induced self-disassembled endo/lysosome pH-responsive, providing their disassembly, and controlling encapsulated DOX [77].

Heparin, an inhibitor of serine proteases in blood coagulation, is also used in antitumor chemotherapy due to its ability of inhibiting tumor growth and metastasis. Based on those properties a novel drug delivery system to carry DOX containing heparin dendronized was designed and synthesized via click chemistry. An acid-labile hydrazone bond was employed for breast tumor therapy. Dendronized heparin-DOX conjugate was not toxic, comparatively to free DOX in histological analysis. Additionally, the dendronized derivative demonstrated high antitumor activity on breast cancer cell line, as well as antiangiogenics effects and apoptosis induction. According to the She and colleagues work [78], this conjugate may not only be a background for safe nanoparticles design but also an efficient carrier for drug delivery.

**Figure 13:**
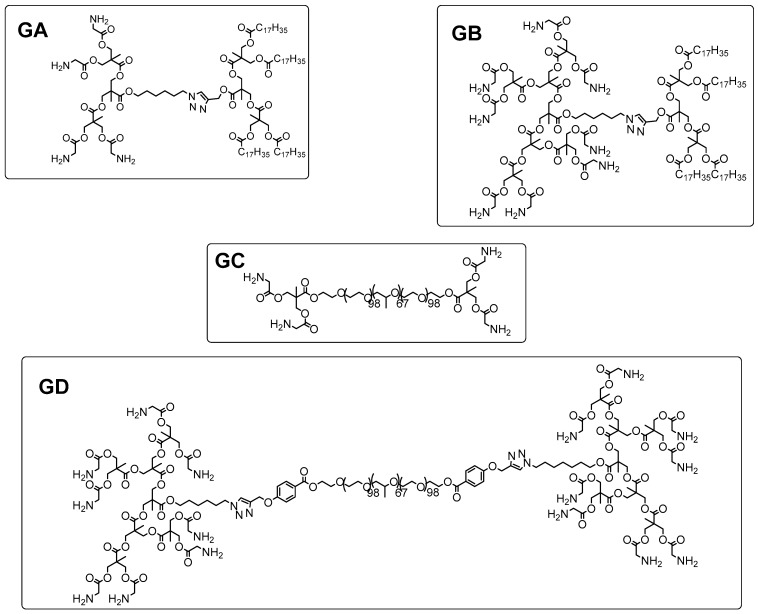
Janus dendrimer (with two different generations GA and GB) and hybrid dendritic-linear-dendritic block copolymers (with two different generations GC and GD) to of CQ, PQ and rhodamine B encapsulation [79].

An alternative to PAMAM dendrimers for drug delivery via encapsulation was developed using other polymeric structures. Although PAMAM dendrimers with antimalarial drugs exhibited specific binding, their IC_50_ were modest against *Plasmodium*-infected cells. Taking this into account, a Janus dendrimer (with two different generations GA and GB—Figure 13 and hybrid dendritic-linear-dendritic block copolymers (with two different generations GC and GD—Figure 13) were synthesized, with three encapsulated drugs (chloroquine-CQ, primaquine-PQ and rhodamine B) against *Plasmodium falciparum*. In vitro tests showed better efficacy of the conjugate when compared with free CQ. However, in vivo assays have shown no drug efficacy improvement when GD-CQ is compared with CQ. In both cases, the mice survival was slightly better for GD-CQ and GC-PQ dendrimers (Figure 13) [79].

Dendrimers composed of fluocinolone acetonide were designed to treat neuroinflammation in the outer retina, when coupled to G4-OH PAMAM through the spacer glutaric acid. Conjugated dendrimer labeled to fluorescein isothiocyanate (label to cell uptake visualization) presented higher uptake than free-fluorescein isothiocyanate according to Royal College of Surgeons retinal degeneration rat models. Iezzi and coworkers [80] used dendrimers conjugated to Cy5.5-mono-NHS ester (another labeled non-susceptible to tissue autofluorescence) to explain that effect and observed the same profile mentioned above, in which, after 35 days of administration, the dendrimers were still present in target cells. Additionally, the dendrimer containing fluocinolone acetonide showed better performance than the free drug in attenuation neuroinflammation and neuroprotection. According to the authors, dendrimer cell uptake was enhanced, increasing the drug residence time, delivering specific retinal area, and reducing side effects due to PAMAM dendrimers intrinsic ability to their localization within activated microglia.

## 2. Concluding Remarks

Selectivity has been the goal of chemotherapeutic agents, mainly for cancer. That is why most papers herein presented are related to tumor targeting. It is interesting that most of the examples are referred to doxorubicin (DOX) conjugation, probably because it has been used for many kinds of tumors and its severe side effects not rarely compromise its therapeutic application. Besides DOX, methotrexate (MTX) is the prototype in the design of selective conjugate compounds. On the other hand, dendrons and dendrimers, as well, are interesting kind of polymers whose properties favor their use either to attach covalently or to encapsulate bioactive compounds giving prodrugs and delivery forms of drugs, respectively. Those carriers are very flexible, in terms of positions they furnish for bonding different molecules, including target groups. Using dendrons and dendrimers it is possible to achieve selective delivery, provide the specific and proper target group is chosen.

Different kinds of targeting groups have been used and this is possible due to the advance in the study of molecular biology and genetics, which allows the discovery of cell receptors and selective mechanisms of drug release. Obtaining selective dendrimer conjugate drug compounds has been a complex goal, but the interesting properties the matrix imparts in terms of toxicity, solubility, bioavailability, and effectiveness, among others, compensates for the complexity mentioned before.

Although cancer has shown to be one of the main cases of death worldwide, it is important to think about other classes of diseases, as the neglected ones. This review shows few examples of application of the approach of targeting drugs by means of dendrons or dendrimers for those diseases. The reason probably is the low interest the research on this kind of drugs arouses in general, which leads to the thinking that the complexity, besides the costs, do not compensate the low revenues for pharmaceutical industries.

Our team has been using dendrons and dendrimers and some selective groups based on specific cell receptors to obtain target drugs (data not published). We have been working on neglected diseases and mainly for Chagas disease, leishmaniasis and malaria our goal is to obtain dendrons and/or dendrimers through prodrug design using drugs and/or bioactive compounds. We intend to stimulate research groups working either on target dendrons or dendrimers to apply their ideas to obtain specific conjugate compounds for neglected diseases. It is worth noting that the 17 diseases that the World Health Organization (WHO) considers neglected ones are responsible for 1 billion people infected worldwide [117].

## Figures and Tables

**Table 1:** Some targeting groups used for dendrons and dendrimers.

Directing Group	Disease	Dendrimer	Results	References
Peptides	Cancer	PAMAM	The RGD modified dendrimer showed a higher therapeutic effect on melanoma cells and a higher accumulation in tumor regions	[41]
	Cancer	PAMAM	The modified PAMAM dendrimer showed a selective intake in melanoma cells. However, showed a low tumor intake	[42]
	Cancer	Janus	The modified dendrimer showed an increased targeting property and optimized release property	[43]
	Cancer	PPI dendron	The modified dendron showed a significantly higher cellular uptake and selectivity for lysosomes	[44]
	Cancer	PAMAM	Higher in vitro uptake and in vivo accumulation	[45]
	Cancer	PEG	The dendrimer showed an excellent load capacity and synergic effect of both substituents in vivo and in vitro	[46]
	Cancer	PAMAM	The modified dendrimer showed a greater cellular uptake of 5-FU	[47]
	Cancer	PLL	The dendrimer showed a high cellular uptake and could be carried into lysosomal compartments	[48]
	Cancer	Substance P dendron	The SP dendron showed a higher cellular uptake and decreased tumor cell viability	[49]
	Cancer	PAMAM	The (GFLG) dendrimer–DOX was more accumulated in tumor area than in liver and other organs	[50]
	Cancer	PLL dendron	The dendritic drug delivery system showed better biosafety and higher in vitro cytotoxicity	[51]
	Cancer	PLL dendron	The modified dendrimer demonstrated targeting ability at both in vitro and in vivo assays, also it exhibited tumor growth inhibition	[52]
	Cancer	DendGDP	The conjugate dendrimers presented superior cell uptake than free DOX in vitro trials	[3]
Folate	Cancer	PPI	The modified dendrimer showed lower toxicity and higher cellular uptake	[53]
	Cancer	PAMAM	The modified dendrimer showed higher tumor cell cytotoxicity	[54]
	Cancer	PAMAM	The FA modified dendrimer showed a lower healthy cell toxicity and higher cancer cell accumulation	[55]
	Cancer	PAMAM	The FA modified dendrimer showed a better activity against tumor cells	[56]
	Cancer	PAMAM	The designed G5 PAMAM coupled to MTX and FA was more efficient and presented higher action in tumor cells	[57]
	Cancer	PAMAM	The modified dendrimer showed a lower toxicity and increased half-life	[58]
	Cancer	PAMAM	There was not a difference in the activity between the G3 and G5 dendrimer. Both showed a good delivery system for the drug	[59]
	Cancer	PAMAM	The dendrimer improved the solubility of the flavonoid and showed a high selectivity for HeLa cells	[60]
	Cancer	PAMAM	The dendrimer showed a high accumulation on tumor sites, which indicates a promising use as drug delivery and diagnostics	[61]
	Arthritis	PAMAM	The dendrimer showed a higher plasma concentration, higher selectivity, and lower gastric toxicity	[62]
	Arthritis	PAMAM	The indomethacin-FA-dendrimer showed a more controlled release than other dendrimers	[63]
	Cancer	PAMAM	These dendrimers showed high loading capacity, low cytotoxicity, and redox-driven cleavage through disulfide bridges	[64]
Carbohydrates	Cancer	PAMAM	The conjugated dendrimers showed a much higher HepG2 uptake than the non-conjugated	[65]
	Malaria	PPI	The galactose conjugated dendrimer was able to decrease the hemolytic property of the primaquine	[66]
	HIV	PPI	Dendrimers were able to decrease the drug toxicity. However, the mannose derivative presented 12-times-higher cellular uptake when compared with that free drug	[67]
	HIV	TPPI	Both dendrimers showed good results in cell uptake assays, since mannose interacted with lectin receptor and TPPI was absorbed via phagocytosis	[67]
	Cancer	Arginine dendron	In vitro assays exhibited excellent biocompatibility. LP-g-G3P/DOX was internalized into the hepatoma carcinoma cells, inhibiting cell proliferation	[68]
	Cancer	PPI	The dendrimer exhibited lower hemolytic property than free drug and a better accumulation in the brain than in other organs, such as liver and kidney	[69]
Monoclonal antibodies	Cancer	PAMAM	The modified dendrimer was capable of selectively bind to the prostate specific membrane antigen receptor	[70]
	Cancer	PAMAM	This molecule presented high affinity for HER, which resulted in significant internalization of IL-6-G5 PAMAM dendrimers into HeLa cells	[1]
Other Targeting groups	Cancer	PAMAM	The uptake and selectivity in HeLa cells were more appropriate for biotinylated dendrimers and more selective for cancer cells	[71]
	Cancer	PAMAM	The dendrimer showed better cellular uptake profile than labeled dendrimer, mainly by respective receptors	[72]
	Cancer	PAMAM	The dendrimer system is a promising nanocarrier because it provides great drug encapsulation, high targeting, and fast drug release in tumor	[73]
	Cancer	PAMAM	The PEGylated dendrimer increased cytotoxicity in murine model of B16 melanoma cells and higher free drug concentration in the tumor and greater anticancer action	[41]
	Cancer	PEG dendron	The dendrimer demonstrated higher stability, lower toxicity, greater intracellular uptake, prolonged half-life time, improved biodistribution and enhanced anticancer potency	[74]
	Cancer	PEG	This dendrimer reduced the cell viability and uptake, showing efficient inhibition and accumulation	[75]
	Cancer	PAMAM	The dendrimer showed a higher inhibitory effect in the in vivo tests and a higher release rate	[76]
	Cancer	PLL	The dendrimer enhanced tumor volume control, permeability, retention effects and heart toxicity, when compared to DOX	[51]
	Cancer	Peptide dendron	This dendron showed an excellent biocompatibility exhibiting pH-responsive, providing their disassembly and controlling encapsulated DOX	[77]
	Cancer	Dendronized heparin	The dendronized derivative demonstrated high antitumor activity on breast cancer cell line, as well as antiangiogenics effects and apoptosis induction	[78]
	Malaria	PAMAM	The dendrimer prodrug showed a better IC50 values however in vivo results showed no difference	[79]
	Neuroinflammation	PAMAM	The dendrimer cell uptake was enhanced, increasing the drug residence time, delivering specific retinal area, and reducing side effects	[80]

## Abbreviations

2-ME	2-methoxyestradiol
5-FU	5-fluorouracil
ASGPR	Asialoglycoprotein receptors
Aspn	Poly aspartic acid
CD	β-cyclodextrin
CMCS	Carboxymethyl chitosan
CPMV	Cowpea mosaic virus
CPPI	PPI dendrimer conjugated with concanavalin A
CQ	Chloroquine
CLNS	Supramolecular dendritic system composed of PLL and poly(l-leucine)
DOTA	1,4,7,10-tetraazacyclododecane-1,4,7,10-tetraacetic acid
DOX	Doxorubicin
EPR	Enhanced permeability and retention effect
FA	Folic acid
FKE	Tripeptide Phe-Lys-Glu
Gd III	Gadolinium
GFLG	Tetrapeptide Gly Phe-Leu-Gly
HAP	Hydroxyapatite
HER	Human epidermal growth factor receptor
IL-6	Interleukin-6
MPPI	Mannosylated-PPI
MTX	Methotrexate
NAcGal	*N*-acetylgalactosamine
PAMAM	Poly(amidoamine) dendrimer
PEG	Polyethylene glycol
PEHAM	Poly(etherhydroxylamine) dendrimer
PLL	Poly(lysine) dendrimer
PLLD	Poly(l-lysine) branch
PPCD	PEG-PAMAM-*cis*-aconityl-DOX conjugates
PPI	Poly(propylenimine) dendrimer
PPSD	PEG-PAMAM-succinic-DOX conjugates
PQ	Primaquine
PVGLIG	Hexa-peptide Pro-Val-Gly Leu-Ile-Gly
RGD	Tripeptide Arg-Gly Asp
SP	Substance P
SPION	Superparamagnetic iron oxide nanoparticle core
SPPI	PPI dendrimer functionalized with sialic acid
Tf	Transferrin
TPPI	T-Boc-glycine-PPI
UA	Ursolic acid
WGA	Wheat germ agglutinin
